# Candidate Markers of Olaparib Response from Genomic Data Analyses of Human Cancer Cell Lines

**DOI:** 10.3390/cancers13061296

**Published:** 2021-03-15

**Authors:** Setor Amuzu, Euridice Carmona, Anne-Marie Mes-Masson, Celia M. T. Greenwood, Patricia N. Tonin, Jiannis Ragoussis

**Affiliations:** 1 Department of Human Genetics, McGill University, Montreal, QC H3A 0C7, Canada; celia.greenwood@mcgill.ca (C.M.T.G.); patricia.tonin@mcgill.ca (P.N.T.); ioannis.ragoussis@mcgill.ca (J.R.); 2 McGill Genome Centre, McGill University, Montreal, QC H3A 0G1, Canada; 3 Centre de Recherche du Centre Hospitalier de l’Université de Montréal (CRCHUM), Montreal, QC H2X 0A9, Canada; euricarmona@gmail.com (E.C.); anne-marie.mes-masson@umontreal.ca (A.-M.M.-M.); 4 Institut du Cancer de Montréal, Montreal, QC H2X 0A9, Canada; 5 Department of Medicine, Université de Montréal, Montreal, QC H3C 3J7, Canada; 6 Lady Davis Institute for Medical Research, Jewish General Hospital, Montreal, QC H3T 1E2, Canada; 7 Departments of Oncology and Epidemiology, Biostatistics and Occupational Health, McGill University, Montreal, QC H3A 1A2, Canada; 8 Cancer Research Program, Centre for Translational Biology, The Research Institute of the McGill University Health Centre, Montreal, QC H4A 3J1, Canada; 9 Department of Medicine, McGill University, Montreal, QC H3A 0G4, Canada

**Keywords:** olaparib, genomic markers, PARP1, cancer cell lines

## Abstract

**Simple Summary:**

Olaparib is an oral medication typically used to treat certain advanced ovarian and breast cancers with mutations in *BRCA1* or *BRCA2* genes. Mutations in these genes can increase the risk of developing breast, ovarian, and other types of cancer. Olaparib is the first clinically approved drug that specifically targets a vulnerability of cancers with these mutations. Genetic alterations in cancer tumors can affect response to treatment in cancer patients. Cancer models such as cell lines, which are cancer cells derived from patients and have been grown in the laboratory over time, can be used to identify these alterations which may contribute to sensitivity or resistance to treatment. We analyzed data from two independent groups of cancer cell lines and identified alterations in additional genes (*PUM3*, *EEF1A1* and *ELP4*) that potentially increase sensitivity to olaparib. Further experimental and clinical investigations are required to validate our findings.

**Abstract:**

The benefit of PARP inhibitor olaparib in relapsed and advanced high-grade serous ovarian carcinoma (HGSOC) is well established especially in *BRCA1/2* mutation carriers. Identification of additional biomarkers can help expand the population of patients most likely to benefit from olaparib treatment. To identify candidate markers of olaparib response we analyzed genomic and in vitro olaparib response data from two independent groups of cancer cell lines. Using pan-cancer cell lines (*n* = 896) from the Genomics of Drug Sensitivity in Cancer database, we applied linear regression methods to identify statistically significant gene predictors of olaparib response based on mRNA expression. We then analyzed whole exome sequencing and mRNA gene expression data from our collection of 18 HGSOC cell lines previously classified as sensitive, intermediate, or resistant based on in vitro olaparib response for mutations, copy number variation and differential expression of candidate olaparib response genes. We identify genes previously associated with olaparib response (*SLFN11*, *ABCB1*), and discover novel candidate olaparib sensitivity genes with known functions including interaction with PARP1 (*PUM3*, *EEF1A1*) and involvement in homologous recombination DNA repair (*ELP4*). Further investigations at experimental and clinical levels are required to validate novel candidates, and ultimately determine their efficacy as potential biomarkers of olaparib sensitivity.

## 1. Introduction

Olaparib is the first poly(adenosine diphosphate[ADP]-ribose) polymerase (PARP) inhibitor to be clinically approved as maintenance therapy for treatment of advanced or recurrent ovarian cancer. It inhibits PARP1 and PARP2 which are involved in the base excision repair (BER) pathway important for repair of damaged bases and single-strand DNA breaks (SSB). The antitumor activity of olaparib is based on the synthetic lethality relationship between PARP and BRCA1/2 where loss of BRCA1/2 function or PARP inhibition alone is compatible with cell survival, but the combination of BRCA1/2 inactivation and PARP inhibition leads to cell death [1]. Olaparib and other PARP inhibitors (PARPis) are especially cytotoxic to *BRCA1/2*-mutated, homologous recombination (HR) DNA repair deficient, tumor cells by blocking PARP-mediated DNA repair and promoting DNA replication stress through trapped PARP-DNA complexes leading to chromosomal instability, cell cycle arrest and ultimately apoptosis [2,3,4]. Olaparib treatment has been most successful in minimizing tumor growth and delaying tumor recurrence in high-grade serous ovarian carcinoma (HGSOC) [5,6], the most common and most lethal subtype of ovarian cancer, where 50% of cases are estimated to be HR-deficient primarily through genomic inactivation of *BRCA1/2* [7,8]. Pathogenic variants of *BRCA1* and *BRCA2*, occurring at germline or somatic levels, are major genetic risk factors for HGSOC and are associated with about 20–25% of HGSOC cases [7,9,10,11]. While initial response rates to standard first-line chemotherapy, consisting of cytoreductive surgery and platinum-based chemotherapy, for HGSOC are high (>70%), disease recurrence is also correspondingly high [12]. PARP inhibitors have emerged as promising maintenance therapy for patients with *BRCA1/2*-mutated HGSOC who were initially sensitive to platinum-based chemotherapy (PBC). Indeed, *BRCA1/2* mutations and alterations in other genes that sensitize tumors to PBC also sensitize tumors to PARP inhibitors, as we have recently reviewed [13].

Genomic and molecular alterations in HR repair genes and genes of related pathways such as the Fanconi anemia (FA) pathway [14], and genes in other DNA repair pathways including BER [15,16], mismatch repair (MMR) and nucleotide excision repair (NER) [17], DNA replication fork protection [18,19], and cell cycle regulation [20,21] have been associated with olaparib sensitivity and resistance mainly through in vitro analysis of human cancer cell lines.

We sought to identify new genomic markers of olaparib response beyond these pathways given that PARP1, the most abundant and most active PARP enzyme, has been reported to have other roles beyond DNA repair such as transcription [22], inflammation [23], and angiogenesis [24,25], suggesting that response to PARP inhibition may be influenced by other factors involved in these additional roles. To discover new olaparib response genes, we analyzed publicly available in vitro olaparib response and mRNA gene expression data from pan-cancer cell lines in the Genomics of Drug Sensitivity in Cancer (GDSC) [26] database to find genes whose expression significantly predicts olaparib sensitivity or resistance using multivariate and univariate linear regression methods. Our analysis identified known olaparib response genes as well as novel candidate genes. We then validated these candidate genes by identifying genomic alterations involving these genes in 18 HGSOC cell lines that we previously classified into sensitive (*n* = 5), intermediate (*n* = 9), and resistant (*n* = 4) groups based on in vitro olaparib response [17]. These cell lines are long-term passages derived from tumor or ascites of HGSOC cases that were treatment-naïve or treated with PBC and have been extensively characterized at genetic and molecular levels and reflect some of the features of HGSOC cases including *BRCA1/2* and *TP53* mutations [27,28,29,30]. Candidate genes derived from analyses of GDSC cell lines were investigated for protein-coding and splice site sequence variants, copy number variations, and differential expression between sensitive and resistant HGSOC cell lines. In this research article, we present our analyses, findings and hypotheses for how key validated genes, including PARP1 interactors and emerging HR genes, may mediate olaparib response and therefore warrant further investigations as potential biomarkers of olaparib response.

## 2. Results

Univariate and multivariate linear regression methods were used to estimate the relationships between basal mRNA gene expression and olaparib response (IC_50_) in 896 human-derived cell lines from diverse types of cancer. This approach revealed 83 significant gene predictors in common from 121 multivariate and 1176 univariate significant gene predictors. The complete list of candidate genes from both analyses are in Table S1 (multivariate), Table S2 (univariate), and Table S3 (common predictors). In total, there are 1214 unique, significant gene predictors. These candidate olaparib response genes are either associated with increased sensitivity or increased resistance. Among the candidate genes, 33 are known to be involved in DNA repair or cell cycle regulation pathways (Table 1). These include *APTX* which is involved in single strand break repair. Expression of *APTX* is associated with increased sensitivity to olaparib. *E2F1* expression was associated with olaparib resistance from both multivariate and univariate analyses. E2F1 is a transcription factor that promotes expression of several DNA repair genes including HR genes *BRCA1* and *RAD51* [31,32]. Cyclin dependent kinase inhibitors *CDKN2A*, *CDKN2B*, and *CDKN2C* were found to be associated with resistance. Expression of *TP53*—a key regulator of genomic stability, cell proliferation and death—was associated with olaparib sensitivity. Expression of *FANCE*, *XRCC5*, and *PMS1* which are involved in FA, non-homologous end-joining, and MMR pathways, respectively, was associated with increased sensitivity to olaparib.

**Figure 1:**
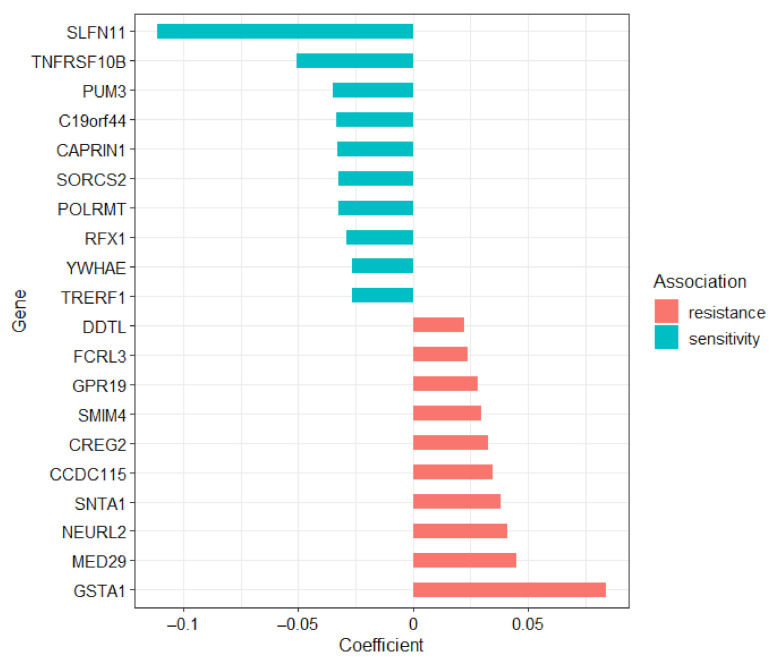
Top 10 gene predictors of olaparib resistance and sensitivity from linear regression analyses of GDSC pan-cancer cell lines. Coefficients from elastic net multivariate linear regression are shown on the horizontal axis and gene symbols on the vertical axis. Genes associated with resistance have coefficients greater than zero (increased IC_50_), while genes associated with sensitivity have coefficients less than zero (decreased IC_50_).

The top 10 genes associated with sensitivity or resistance to olaparib among the common predictors are shown in Figure 1.

### 2.1. Known Markers of Olaparib Response Are among Candidate Olaparib Sensitivity and Resistance Genes

*SLFN11* expression was most strongly associated with increased sensitivity to olaparib among common predictors. *SLFN11* expression was found to be correlated to PARP inhibitor response, especially talazoparib, in the NCI-60 panel of human cancer cell lines and was experimentally shown to sensitize cancer cells to PARP inhibitors including olaparib [21]. Second among the top common predictors of sensitivity, *TNFRSF10B* also known as Death Receptor 5 (DR5) has also been previously associated with PARPi response and is highly expressed in sensitive cells [33]. On the other hand, among resistance candidate genes, *GSTA1* mRNA expression is the top predictor of resistance to olaparib among common predictors and has been found to be involved in cisplatin resistance [34]. Outside of the top candidates above, other genes from these analyses have also been reported to be associated with olaparib response. For example, ATP Binding Cassette Subfamily B Member 1 (*ABCB1*), associated with resistance from univariate analysis, encodes MDR1 a P-glycoprotein drug efflux pump that has been linked to resistance to olaparib and chemotherapeutic agent paclitaxel [35,36]. Additionally, Ubiquitin Conjugating Enzyme E2 R2 (*UBE2R2*) was associated with sensitivity to olaparib from both univariate and multivariate analyses and was previously identified as a candidate olaparib sensitivity gene in complementary RNA interference screens [37].

### 2.2. Novel Candidate Markers of Olaparib Response

**Figure 2:**
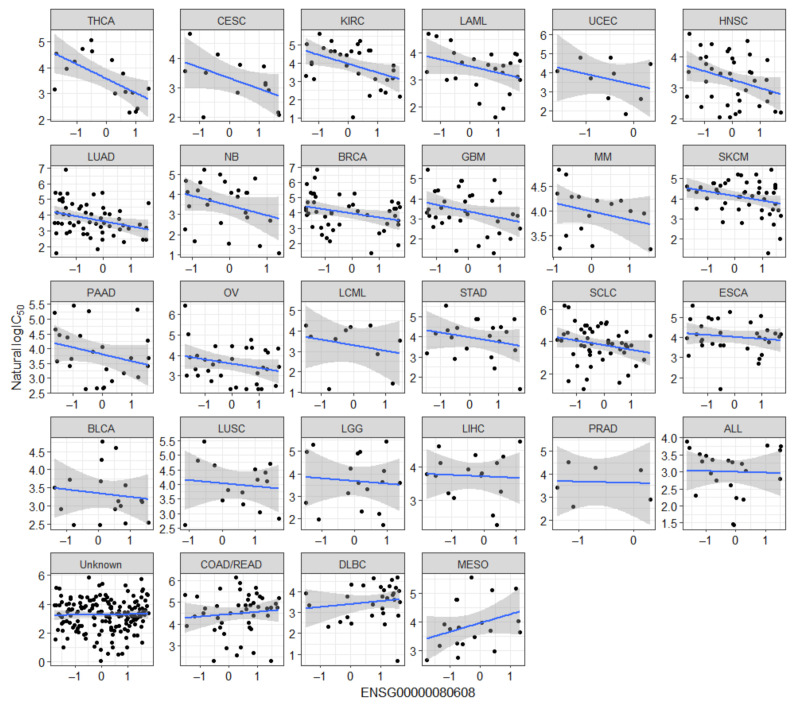
Correlations between *PUM3* mRNA expression and Olaparib IC_50_ in GDSC cell lines of multiple cancer types. Each plot shows *PUM3* z-score expression (horizontal axis) plotted against natural log of olaparib IC_50_ (vertical axis) in a specific cancer type (in the title) based on TCGA classes. Plots are arranged from top left (THCA: Thyroid carcinoma) to bottom right (MESO: Mesothelioma) in order of increasing Pearson correlation coefficient. Cell lines of unknown cancer type are also shown in the plot titled Unknown. Complete list of abbreviations in plot title and meaning is in Table 3 of Materials and Methods. Notable cancer types where olaparib treatment is approved—OV (Ovarian serous cystadenocarcinoma), BRCA (Breast invasive carcinoma) are shown. Blue lines in each plot are linear regression lines. Shaded region around blue lines represent 95% confidence region. Only cancer types with at least five cell lines are shown.

Although both analyses rediscover several known markers of olaparib response, there are also many novel candidates that have not been previously linked, statistically or experimentally, to olaparib response. Pumilio RNA Binding Family Member 3 (*PUM3*) is one of these candidates. *PUM3* is one of the top 10 predictors of olaparib sensitivity (3rd in Figure 1) and was identified by both multivariate and univariate analyses (Table 2). *PUM3* mRNA expression is negatively correlated with olaparib IC_50_ in 20 cancer types (Figure 2) with Pearson’s r ranging from −0.63 to −0.11. PUM3 is known to interact with PARP1 by binding to its catalytic domain and inhibiting its poly ADP-ribosylation activity [38]. This is relevant because olaparib also binds to the catalytic domain of PARP1 to inhibit PARP1 catalytic activity. Suggesting that PUM3 may act as a potential endogenous inhibitor of PARP1, at least in some context. 

Like *PUM3*, *EEF1A1*, is another gene encoding a protein that interacts with PARP1 and emerged as a significant predictor of olaparib sensitivity. *EEF1A1* encodes eukaryotic translation elongation factor 1 alpha 1 which is a subunit of elongation factor complex 1 and is involved in protein synthesis where it promotes binding of aminoacyl-tRNA to ribosomes in a guanosine triphosphate (GTP)-dependent manner [39]. It also forms a complex with PARP1 and tyrosine protein kinase TXK to function as a T-helper 1 (Th1) cell-specific transcription factor, that binds to the promoter of interferon gamma (IFNG) and is therefore involved in Th1 cytokine production [40]. From the univariate analysis, expression of *EEF1A1* was associated with increased sensitivity to olaparib (Table 2). Although EEF1A1 interacts with PARP1 it has also not been previously linked to PARP inhibitor response. Upregulation of *EEF1A1* has been reported to have pro-apoptotic effect [41]. EEF1A1 is also known to be involved in cytoskeletal organization and cell morphology through interaction with actin [42,43].

Elongator Acetyltransferase Complex Subunit 4, *ELP4*, is another interesting candidate olaparib sensitivity gene that emerged as a significant predictor from the univariate analysis. From this analysis, *ELP4* and *ELP5* mRNA expression were significantly associated with increased sensitivity to olaparib (Table 2). ELP4 and ELP5 are subunits of the elongator complex (comprised of ELP1, ELP2, ELP3, ELP4, ELP5, ELP6) whose functions include transcriptional elongation [44], and tRNA modification [45]. Notably, ELP4 was reported to be a novel HR repair pathway gene [46]. 

### 2.3. Characterization of Sequence and Copy Number Variation from Whole Exome Sequencing and Differential Gene Expression Analysis of HGSOC Cell Lines

**Figure 3:**
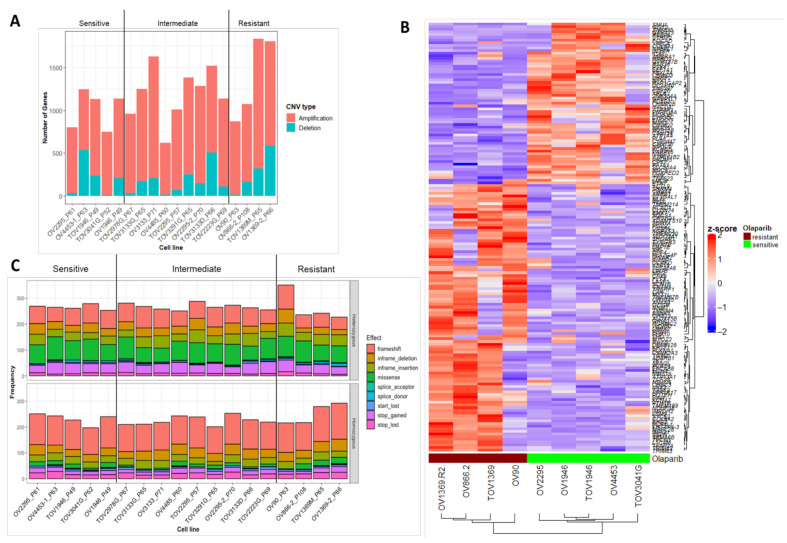
Sequence variants, copy number variation, and differential gene expression analysis of HGSOC cell lines. (**A**). Number of genes involved in copy number amplifications and deletions per HGSOC cell line. From left to right, cell lines are arranged from most sensitive (OV2295) to most resistant [OV1369(R2)] to olaparib. (**B**). Heatmap of significant differentially expressed genes between sensitive and resistant HGSOC cell lines. (**C**). Frequency of rare protein-coding or splice-site, heterozygous (top) and homozygous (bottom), sequence variants predicted to be functionally damaging or deleterious using in silico tools. Cell lines arranged, left to right, in order of increasing olaparib resistance. OV2295 and OV1369(R2) are most sensitive and resistant cell lines, respectively.

HGSOC cell lines that we previously classified as sensitive, intermediate, resistant to olaparib [17] were characterized in terms of sequence (SNVs, indels) and copy number variation using whole exome sequencing (WES) data, and differentially expressed genes using mRNA gene expression microarray data (Figure 3). CNVs were found to be prevalent in the HGSOC cell lines. On average, 989 genes are amplified, and 201 genes are deleted per cell line (Figure 3A). The total number of unique genes that are amplified or deleted across the cell lines are 4581 (67%) and 2258 (33%), respectively. Similarly, more genes are amplified than deleted among epithelial ovarian cancer (EOC) cases (*n* = 572) in the TCGA PanCancer Atlas 2018—22,235 (58%) and 16,419 (42%), respectively. Copy number amplification of *CCNE1* locus was observed in two HGSOC cell lines, OV866(2) (resistant) and TOV3291G (intermediate), as previously reported (Figure S1) [29]. *CCNE1* is amplified in approximately 20% of HGSOC cases [7]. *MYC* is amplified in intermediate cell lines TOV2295(R) and TOV2978G. Other oncogenes, MECOM and KRAS were also found to be amplified in intermediate [OV2295(R2), OV3133(R), TOV223G, TOV3133D, TOV3133G] and resistant [OV866(2), OV1369(R2), TOV1369] cell lines, respectively. Similarly, MYC (33.2%), MECOM (27.8%), and KRAS (9.4%) are amplified in EOC cases of the TCGA PanCancer Atlas [47,48] dataset.

In total 162 genes were significantly differentially expressed (Figure 3B) between resistant and sensitive cell lines, with 45 (27.8%) of these genes involved in CNVs through amplifications and deletions. Protein-coding and splice-site sequence variants were investigated for all HGSOC cell lines. The frequency of rare (less than 0.1% minor allele frequency in gnomAD database) variants predicted to be functionally damaging or deleterious using in silico prediction tools are presented for each HGSOC cell line in Figure 3C. On average, there are 498 functionally relevant homozygous and heterozygous sequence variants per cell line with range from 453 to 567. Correspondingly, the average number of mutated genes per cell line is 424 with range from 375 to 484. Notable mutations in *TP53* and *BRCA1/2* in these cell lines were rediscovered [28,29,30,49]. All but one (TOV3041G) of the cell lines is mutated in *TP53*. OV4485 and OV4453 harbor pathogenic variants in *BRCA1* (c.4548-1G>T, splice acceptor) and *BRCA2* (p.Glu1953 *, stop-gained) of germline origin (Figure S2). 

Finally, analysis of single base substitution (SBS) mutational signatures in the 18 HGSOC cell lines revealed that cell lines exhibit multiple COSMIC (catalogue of somatic mutations in cancer) mutational signatures. The dominant signatures are 1 and 3, which are associated with aging and HR repair deficiency, respectively (Figure S3). These are also the dominant signatures that have been reported in EOC cases [50] and show that cell lines are similar to EOC cases based on mutational signatures. *BRCA1/2*-mutated cell lines (OV4485, OV4453) were found to have mutational signature 3.

### 2.4. Novel Candidate Olaparib Response Genes Linked to Genomic Alterations in Independent HGSOC Cell Lines

**Figure 4:**
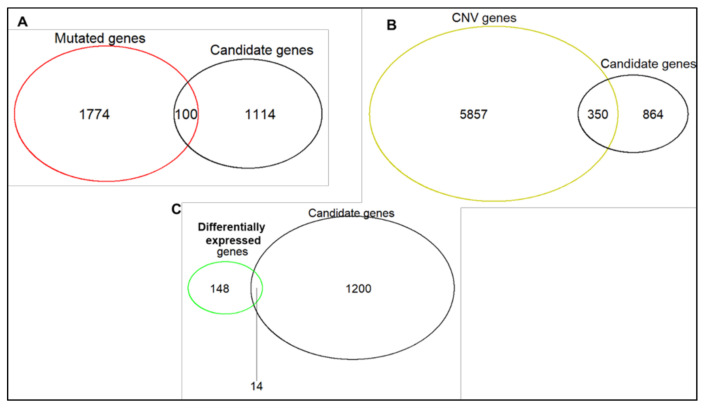
Candidate olaparib response genes derived from GDSC cell lines that are validated in HGSOC cell lines. Number of candidate olaparib sensitivity and resistance genes identified from multivariate and univariate linear regression analyses that are (**A**) Heterozygous or homozygous for rare functionally relevant sequence variants, (**B**) Involved in CNVs (amplifications and deletions), and (**C**) Significantly differentially expressed between resistant and sensitive HGSOC cell lines.

To validate the findings from GDSC pan-cancer cell lines in HGSOC cell lines, all 1214 candidate genes, from univariate and multivariate analyses, were investigated for mutations, CNVs, or whether they were differentially expressed between sensitive and resistant HGSOC cell lines (Figure 4). A total of 431 (35.5%) unique genes were altered in at least one of these ways.

Some validated genes were found to have relevant known functions, such as interactions with PARP1 and involvement in HR, and were prioritized. Genomic alterations involving candidate olaparib response genes identified in olaparib-sensitive or -resistant HGSOC cell lines suggests a role for these candidate genes in olaparib response. Known functions of these validated candidate genes could provide clues for plausible mechanisms by which they mediate olaparib sensitivity or resistance. Key candidate genes with relevant functions and altered in the HGSOC cell lines are presented below. 

**Figure 5:**
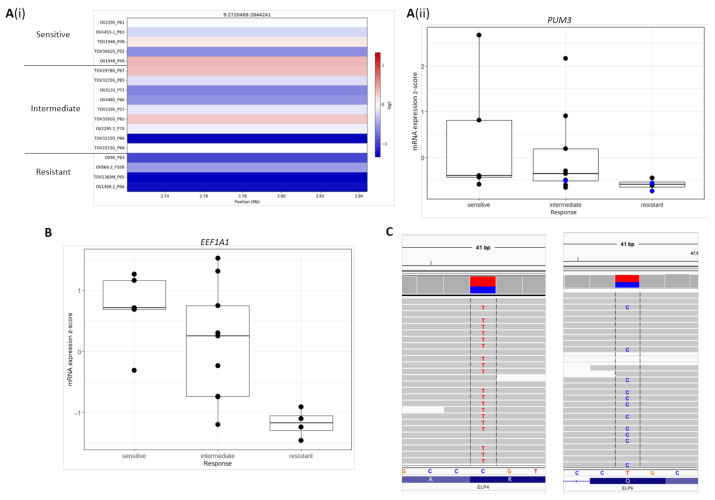
Genomic alterations of *PUM3, EEF1A1, ELP4*, and *ELP5* in HGSOC cell lines. A. *PUM3* copy number profile and mRNA expression in HGSOC cell lines. (**A**(i)). Log2 copy number ratio for copy number segments spanning *PUM3* gene locus for 18 HGSOC cell lines. (**A**(ii)). Boxplots of z-score *PUM3* mRNA expression in sensitive, intermediate, and resistant HGSOC cell line groups. (**B**). *EEF1A1* mRNA expression in HGSOC cell lines in sensitive, intermediate, and resistant olaparib response groups. (**C**). Integrative Genomics Viewer (IGV) screenshots of missense variants in *ELP4* and *ELP5* genes in intermediate HGSOC cell line TOV2978G. Left, *ELP4* missense variant (p.Arg317Cys, rs764805051, 11:g.31669307C>T), read depth—310, VAF—59% (T). Right, *ELP6* missense variant (p.Gln151Arg, 3:g.47539777C>T), read depth—45, VAF—44% (**C**).

Copy number deletions of *PUM3* were found in 2 resistant and 1 intermediate HGSOC cell lines (Figure 5A(i)). Consistent with these copy number deletions, these resistant [TOV1369, OV1369(R2)] and intermediate (TOV3133D) cell lines were also found to express low levels of *PUM3* mRNA compared to sensitive and intermediate cell lines (Figure 5A(ii)). Taken together these findings implicate *PUM3* mRNA expression in olaparib response with high expression associated with increased sensitivity (from GDSC cell lines) and low expression associated with resistance (from HGSOC cell lines). However, some sensitive cell lines also have low expression of *PUM3* suggesting that PUM3 expression alone is not the single predictor of olaparib response. Investigating the HGSOC cell lines for genomic alterations involving *ELP4* revealed rare potentially deleterious, heterozygous, missense variants of *ELP4* (p.Arg317Cys) as well as *ELP6* (p.Gln151Arg) in TOV2978G (Figure 5C). This cell line is in the intermediate response group, it is on the boundary of sensitive and intermediate groups (Table 4) and is reported to be sensitive to carboplatin in vitro [29]. It was not found to have a *BRCA1/2* mutation, or mutations in other canonical HR repair genes but has mutational signature 3. However, our previous work has shown that this cell line does not express *BRCA1* mRNA or protein [29]. While *ELP4* and *ELP5* mRNA expression are associated with olaparib sensitivity from univariate analysis of GDSC pan-cancer cell lines, ELP4 and ELP6 missense variants may contribute to sensitivity to olaparib in the HGSOC cell line TOV2978G.

ELP4 is the only subunit of the elongator complex that has been implicated in HR repair [46]. However, ELP5, and ELP6 may also have roles in HR repair since all three proteins (ELP4, ELP5, and ELP6) share a RecA ATPase-like protein domain, that is also found in RAD51 [51] which plays an important role in homology search and strand exchange in HR and form a discrete subcomplex by dimerization of ELP4/5/6 heterotrimer into a hexameric ring [52,53]. 

*EEF1A1* is a significant differentially expressed gene between olaparib-sensitive and -resistant HGSOC cell lines (Figure 5B). It is highly expressed in sensitive cell lines compared to resistant cell lines. This is consistent with the observation that increased expression of *EEF1A1* is associated with sensitivity to olaparib in the independent GDSC pan-cancer cell lines. 

### 2.5. Frequency of Genomic Alterations Involving Candidate Olaparib Response Genes in the Cancer Genome Atlas (TCGA) EOC Cases

**Figure 6:**
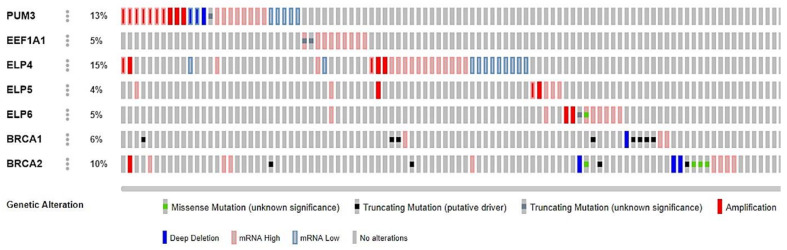
Frequency and types of alterations of key candidate olaparib response genes in TCGA ovarian cancer cases. Mutations, CNVs, and gene expression variation in *PUM3, EEF1A1, ELP4, ELP5, ELP6* compared to *BRCA1* and *BRCA2* in tumor samples (*n* = 201) with complete data from EOC cases in the TCGA PanCancer Atlas study derived from cBioPortal [47,48].

The key candidate genes described above were investigated for mutations, copy number variation, and mRNA expression in patient tumor samples where data for these types of alterations were available (Figure 6). In total, 201 samples from EOC cases in the TCGA PanCancer Atlas dataset were investigated using cBioPortal [47,48]. Notably, high mRNA expression is the most common alteration of *PUM3* in EOC cases and is supported by amplification of *PUM3* in some cases. *PUM3* alterations are almost mutually exclusive of pathogenic variants in *BRCA1* and *BRCA2* (except for one *BRCA1* mutated case). This suggests that a subset of *PUM3*-expressing EOC cases, distinct from *BRCA1/2* mutation carriers, may benefit from olaparib treatment.

## 3. Discussion

The analyses of pan-cancer cell lines in the GDSC database to identify genes whose mRNA expression was significantly associated with sensitivity or resistance to olaparib revealed novel candidate genes with relevant functions. The major findings from this analysis that were successfully validated in the HGSOC cell lines can be classified into two groups of genes: PARP1 interactors (*PUM3*, *EEF1A1*) and emerging HR genes (*ELP4*, *ELP5*, *ELP6*).

PARP1 is the most active target of olaparib [54]. Therefore, any underlying factors that influence PARP1 levels or activity can also affect PARP inhibitor response. *PUM3* mRNA expression was ranked the third strongest predictor of olaparib sensitivity, among common significant predictors from the multivariate and univariate analyses, with increased expression correlated with increased sensitivity in multiple cancer types (Figure 2). *PUM3* (KIAA0020 or human Puf-A, hPuf-A) binds to mRNA and regulates translation using its highly conserved PuF domains. A Puf domain consists of 35 to 39 amino acids capable of associating with the 3′-untranslated region (3′-UTR) of target mRNAs and interacts with other regulatory proteins to promote mRNA degradation and repression of translation [55,56]. Puf proteins are highly conserved among most eukaryotic organisms and are involved in stem cell maintenance, cell development and differentiation. Deletion of PUF-8 in the roundworm, *Caenorhabditis elegans*, led to the development of germ cell tumors [57]. PUM3 is one of the newly discovered members of the human Puf protein family [58]. It shares 63% amino acid homology with zebrafish Puf-A. Unlike classical PUF proteins, which are localized to the cytoplasm PUM3 is predominantly found in the nucleolus. PUM3 has been linked with tumor development. *PUM3* expression has been reported to be positively associated with breast cancer progression. High expression of *PUM3* was observed in 70% of breast cancer biopsies comprising diverse histological subtypes compared to normal breast tissues, ductal carcinoma in situ, and adjacent noncancerous tissues [59]. Downregulation of *PUM3* by siRNA sensitizes cells to the DNA topoisomerase I (TOP1) inhibitor camptothecin and UV treatment, while cells constitutively overexpressing *PUM3* are rendered resistant to genotoxic exposure [38]. However, neither of these DNA damage-inducing agents specifically binds to PARP1 to inhibit PARylation. Cytotoxicity of TOP1 inhibitors is based on interference of DNA replication and transcription by trapped TOP1-DNA cleavage complexes [60,61,62]. UV light can also generate TOP1-DNA cleavage complexes and pyrimidine dimers that impede DNA replication [63,64]. PUM3 interacts with the catalytic domain of PARP1 and inhibits poly(ADP-ribosyl)ation activity of PARP1 in vitro [38]. The effect of *PUM3* gene silencing or deletion on in vitro response to PARPi treatment has not been reported. Our results show that *PUM3* mRNA expression is associated with increased sensitivity to olaparib. This supports the hypothesis that PUM3-mediated inhibition of PARylation by PARP1 may support olaparib-mediated catalytic inhibition of PARP1 and sensitize cells. However, it is not known if PUM3 can contribute to PARP trapping which is considered the major part of PARPi cytotoxicity. 

*EEF1A1* mRNA expression was associated with increased sensitivity to olaparib in the GDSC pan-cancer cell lines and found to be highly expressed in olaparib-sensitive HGSOC cell lines, including *BRCA2*-mutated cell line OV4453, compared to resistant cell lines (Figure 5B). The interaction of EEF1A1 with PARP1 is different from that of PUM3 as it does not involve inhibition of PARylation. EEF1A1 is a subunit of a complex also comprised of PARP1 and tyrosine kinase TXK that functions as a transcription factor for *IFNG* in T-helper 1 cells [40]. *IFNG* expression has been found to be a predictive marker of sensitivity to immune checkpoint inhibitors nivolumab and pembrolizumab in non-small cell lung cancer and melanoma cases, respectively [65]. While it is unclear how *EEF1A1* expression may contribute to olaparib sensitivity, through its interaction with PARP1, EEF1A1 could link PARP inhibition to immunotherapy and may also be a potential marker of sensitivity to immunotherapy or combination of immunotherapy and PARPi. There is interest to combine immunotherapy with PARPis. A phase 2 clinical trial (NCT02734004) of olaparib and programmed cell death ligand 1 (PDL-1) inhibitor durvalumab (Imfinzi) in platinum-sensitive relapsed germline *BRCA1/2*-mutated ovarian cancer is an example [66]. *BRCA1/2*-mutated, HR-deficient HGSOC is associated with increased neoantigens, tumor-infiltrating lymphocytes (TILs) and favorable prognosis than HR-proficient HGSOC [67]. 

Expression of *ELP4* and *ELP5* at mRNA level is associated with increased sensitivity to olaparib in GDSC pan-cancer cell lines. An HGSOC cell line (TOV2978G) with intermediate response to olaparib has rare, potentially damaging heterozygous variants in *ELP4* (p.Arg317Cys) and *ELP6* (p.Gln151Arg). ELP4, ELP5, and ELP6 are subunits of the RNA polymerase II elongator complex [68]. TOV2978G does not have *BRCA1/2* mutations although it does not express *BRCA1* mRNA or protein [29] and we found that it exhibits COSMIC mutational signature 3, associated with HR deficiency. Furthermore, it has been previously shown to be sensitive to carboplatin [29]. *ELP4* was discovered as a novel HR repair gene through coevolution analysis of 600 species and functional experiments, and was found to have coevolved with *BRCA1* and *BARD1* in plants and mammals [46]. *ELP4* was experimentally associated with the HR repair pathway using two systems. Knockdown of *ELP4* function led to a significant reduction in brood size of *C. elegans* following exposure to ionizing radiation. Defective HR repair pathway can cause germline radiation sensitivity [69]. Using the Direct Repeat-Green Fluorescence Protein (DR-GFP) assay, knockdown of *ELP4* significantly reduced HR efficiency in the osteosarcoma cell line U2OS [46]. However, the specific role of *ELP4* in HR is not known. Since ELP4/5/6 form a discrete subcomplex and share a RecA ATPase-like protein domain [52,53] that is also found in key HR protein RAD51, ELP5 and ELP6 may cooperate with ELP4 in a HR repair role. 

Despite the utility of cell lines for drug development and biomarker discovery there are some limitations of these models. These may include selection of cells in vitro that harbor molecular genetic abnormalities that may not recapitulate the microenvironment and exposure to chemotherapeutic drugs of in vivo settings. Our collection of HGSOC cell lines, derived from long-term passages of ovarian cancer specimens, have been extensively characterized demonstrating that their molecular genetic features are consistent with those found in the original cancer specimens and some have retained the capacity to propagate as three-dimensional structures in vitro and in vivo [28,29,30,49]. Notably *PUM3*, *EEF1A1*, and *ELP4,* identified from our bioinformatics analysis of cell lines could be considered top priorities for further investigations based on support of their molecular functions as reported in the literature. Biological assessment of these candidates using more recently developed preclinical ovarian cancer cell models such as patient-derived organoids and patient-derived xenografts, may yield more accurate information regarding their role in conferring PARPi sensitivity as these models mimic the three-dimensional environment of patient tumor samples. 

Chemotherapy can drive genomic alterations and gene expression in tumor cells that could lead to resistance, and the cell lines derived from these tumors retain these alterations. Information about the chemotherapy status of the GDSC cell lines is not available via the GDSC database. Therefore, we could not systematically investigate whether alterations in our candidate genes were enriched in cell lines derived from cases that had chemotherapy compared to cell lines derived from cases without prior exposure to chemotherapy. However, this information is available for the HGSOC cell lines and mRNA expression of *EEF1A1* in olaparib-sensitive HGSOC cell lines provides a clue that expression of at least one of our key candidate genes may be influenced by chemotherapy. *EEF1A1* expression is lower in TOV3041G, derived from a post-chemo case (Table 4), as compared to expression in the remaining four cell lines derived from pre-chemo cases suggesting that *EEF1A1* expression is reduced following chemotherapy. It is not clear whether alterations in our candidates can explain response to PBC agents such as carboplatin. As *PUM3* and *EEF1A1* both interact with *PARP1,* loss-of-function alterations in these genes may only effect PARPi response. *ELP4* has been shown to be involved in HR repair [46] and in this capacity could also affect sensitivity to carboplatin. The potentially damaging *ELP4* missense variant (p.Arg317Cys) in the HGSOC cell line TOV2978G likely contributes to its sensitivity to carboplatin even though TOV2978G was not found to express *BRCA1* mRNA or protein [29]. Functional assays in appropriate HGSOC-derived model systems focusing on our candidates would be required to demonstrate their role in affecting alterations in sensitivity to PARPi and PBC.

Our findings associate several genes with olaparib response. We identify known olaparib response genes in relevant pathways such as DNA repair and cell cycle control but also discover novel genes that have not been previously associated with olaparib response such as *PUM3* and *EEF1A1* which are known to interact with PARP1, and *ELP4* a recently discovered HR gene.

## 4. Materials and Methods 

### 4.1. Data Description and Linear Regression Analyses

GDSC is a pharmacogenomic database (cancerrxgene.org) providing genomic data for over 1000 cell lines derived from diverse human cancers, and drug response (IC_50_) data for over 300 drugs and compounds [26]. Olaparib response data is derived from GDSC1 release 7.0 (March 2018). Cell line in vitro drug response was measured using fluorescence-based cell viability assays after 72 h of drug treatment. Cell viability reduction in response to olaparib treatment was expressed in terms of IC_50_. Dose response curves were fitted to fluorescence signal intensities using a non-linear mixed effects model [70]. Olaparib response data was downloaded from ftp://ftp.sanger.ac.uk/pub4/cancerrxgene/releases/release-7.0/v17.3_fitted_dose_response.xlsx, accessed on 8 July 2018. GDSC cell lines analysed in this study are presented in Table 3. 

Gene expression data is merged RNAseq data for these cell lines derived from GDSC, Cancer Cell Line Encyclopedia (CCLE) [71], and Genentech [72] which was previously used to investigate transcription factor-drug interactions and reported by Garcia-Alonso et al., 2018 [73]. Data is preprocessed, normalized, batch-corrected, and filtered to remove low expressed genes and samples. This data is available at the following link: https://www.synapse.org/#!Synapse:syn10463688/wiki/463140, accessed on 7 June 2018. In total, 896 Olaparib-screened cell lines with mRNA expression data for 15,379 genes was available for analysis (Figure S4). This formed the working dataset and was analysed using multivariate (all genes analyzed simultaneously) and univariate (genes analyzed one at a time) linear regression method.

In the multivariate approach, data was randomly partitioned into training (60%) and test (40%) sets, ensuring that these partitions were balanced to have similar proportions of cell lines from each tissue type. A linear regression model with elastic net regularization was fit using natural log IC_50_ as response and z-score expression for all genes as predictors with tissue of origin, microsatellite instability (MSI) status (MSI-high: MSI-H, microsatellite stable: MSS), *BRCA1/2* mutation status (encoded as 1 for mutation in either *BRCA1* or *BRCA2,* and 0 for cell line without mutation in either *BRCA1* or *BRCA2*), first two principal components (Figure S5) of gene expression principal component analysis (PCA), and culture medium (RPMI [Roswell Park Memorial Institute] or DMEM/F12 [Dulbecco’s Modified Eagle Medium F-12]) as additional predictor variables. Five-fold cross-validation was performed on the training set over a range of tuning parameters (alpha ranges from 0, 0.5 or 1 and lambda ranges from 0 to 1 with 0.01 increment). The optimum model (alpha = 0.5, lambda = 0.14) was selected based on lowest root mean-squared error (RMSE). 

The performance of the elastic net multivariate model was evaluated on the test data partition (Figure S6). This model explained 25.2% (R^2^) of the variation in natural log IC_50_ with RMSE = 0.898 and mean absolute error (MAE) = 0.734. Genes with coefficients greater or equal to zero were considered significant gene predictors. Data partitioning, model fitting and evaluation, and visualization were done in R using caret [74], glmnet, and ggplot2 packages.

Multiple ordinary least squares (OLS) linear regression models, for one gene at a time, were also done with natural log IC_50_ as response and z-score of gene expression as a predictor, keeping the same additional predictor variables as the multivariate approach. Correction for multiple testing was done using False Discovery Rate (FDR), genes with FDR-adjusted *p*-values less than 0.05 were considered significant gene predictors. This was done in R using functions lm and p.adjust from *stats* package.

### 4.2. Exome Characterization and Differential Gene Expression Analysis of 18 HGSOC Cell Lines Previously Screened for In Vitro Olaparib Response

*In vitro* olaparib response of 18 spontaneously immortalized human HGSOC cell lines (Table 4)⁠, derived from chemo-naïve and chemo-treated patient’s tumors and ascites was determined by clonogenic survival assay and expressed as IC_50_ as previously described [17]. Briefly, cells were incubated with the drug for 24 h. Cell viability was measured by formation of colonies as observed under a stereomicroscope and reported as percent of control. Experiments were done in triplicate and repeated three times. Cell lines were classified as olaparib sensitive (0.0003 ± 0.0004–0.07 ± 0.05), intermediate (0.45 ± 0.30–2.99 ± 1.20) or resistant (7.04 ± 2.33–21.71 ± 10.33) based on statistical groupings of IC_50_. 

These cell lines have been previously characterized at genetic and molecular levels [28,29,30,49]. All but one (TOV3041G) of the 18 cell lines harbor somatic mutations in *TP53*, which is the most common somatically mutated gene in HGSOC cases. However, TOV3041G does not express TP53 at protein level. Two cell lines were derived from patients that carry germline pathogenic variants in *BRCA1* (OV4485) or *BRCA2* (OV4453) [29].

### 4.3. Exome Sequencing, Read Mapping and Variant Calling

Exome sequencing of the cell lines was done using the Illumina HiSeq 2000 platform, following target enrichment with the Roche Nimblegen SeqCap EZ exome v3 kit, at the McGill University and Genome Quebec Innovation Centre (now called McGill Genome Centre). Sequencing was paired-end with average read length of 100 bases. Sequencing adapters were trimmed and trailing low quality (Phred33 score >= Q30) bases were removed using Trimmomatic [75] (version 0.36). Reads were then aligned to human reference genome build GRCh37 using BWA (Burrows Wheeler Aligner) [76]⁠. Picard [77] (version 2.9.0) was used to mark duplicate reads. Local realignment around indels, and base quality score recalibration was done using GATK (Genome Analysis Toolkit) [78] (version 3.5). SAMtools/BCFtools [79] (version 1.3.1) was used for variant calling. Variant effects were then predicted with SnpEff [80] and annotated with dbSNP [81] and COSMIC (Catalogue of Somatic Mutations in Cancer) [82] identifiers using SnpSift [83]. Variant scores and predictions from variant effect prediction algorithms were obtained from the dbNSFP (database of non-synonymous functional predictions) [84] and dbscSNV (database of splice-altering SNVs) [85] databases. These scores and predictions were also annotated using SnpSift. Annotated variants were then exported into R [86] for further filtering and prioritization.

### 4.4. Filtering and Prioritization of SNVs and Indels

Read depth of 10 or greater and variant allele frequency of at least 30% were used as confidence filtering criteria for variants. Cell lines were investigated for mutations in DNA repair and cell cycle genes (*n* = 533, Table S4). DNA repair genes were derived from a curated list of genes reported in a pan-cancer survey of DNA damage repair deficiency in TCGA (The Cancer Genome Atlas) [87], and cell cycle genes were derived from KEGG (Kyoto Encyclopedia of Genes and Genomes) database [88] and Qiagen’s cell cycle gene expression array [89]. Nonsynonymous SNVs were considered damaging or deleterious based on the consensus prediction of at least four (out of seven) variant effect prediction algorithms; SIFT (Sorting Intolerant from Tolerant) [90], PolyPhen2 (Polymorphism Phenotyping v2) [91], FATHMM-MKL [92], Mutation Assessor [93], Mutation Taster [94], REVEL [95], and MetaSVM [96]. Since damaging variants are rare in the general population an additional criterion for selecting potentially damaging SNVs is that they must be present at 0.1% minor allele frequency (MAF) or lower in the Genome Aggregation Database (gnomAD) [97] database version 2.1, or not reported in this database. Potential splice altering variants were selected based on consensus scores (0.6 or greater) of ADA (adaptive boost) and RF (random forest) in the database of single nucleotide variants within splicing consensus regions (dbscSNV) [85]. Indels called by SAMtools that overlap repeats were filtered out using repeatmasker [98] in rtracklayer [99] package in R. Sequence variants that met filtering and prioritization criteria were manually verified using Integrative Genomics Viewer (IGV) [100].

### 4.5. Copy Number Variation Analysis

CNVkit [101] version 0.9 was used to call CNVs using GRCh37-aligned sequence reads in BAM (Binary Alignment Map) format, genomic coordinates of exome capture target regions in a BED (Browser Extensible Data) file, and GRCh37 reference sequence in FASTA format as inputs. Regions of poor mapping based on GRCh37, containing centromeres, telomeres, and highly repetitive sequences were excluded from the analysis using precomputed BED file included in the software package (https://github.com/etal/cnvkit/blob/master/data/access-5k-mappable.grch37.bed, accessed on 11 March 2018). Target regions were grouped into bins of 267 bp size, on average, according to default settings and read depth for these bins were computed. Off-target coverage was also determined for each cell line. Read depth for each sample is median-centered, across bins, and corrected for GC content and repetitive sequence biases. Corrected bin-level coverage was compared to a neutral (or flat) reference which assumes all target and off-target regions are equally covered and diploid. Bin-level copy number ratios were aggregated into segments using the default circular binary segmentation algorithm with low-coverage and outlier bins filtered out. Genes involved in CNV segments were selected using the genemetrics command with minimum absolute log2 copy ratio threshold (−t) of 0.4 (gain ≥ 0.4, loss ≤ −0.4) and minimum number of bins (−m) per gene of 5. Amplifications and deletions were defined by log2 copy ratio thresholds of 1 and −1, respectively.

### 4.6. Differential Gene Expression Analysis

Gene expression profiling was done for all 18 HGSOC cell lines using the Clariom™ S human array. Normalization was done using Signal Space Transformation-Robust Multi array Average (SST-RMA) method. Normalized expression values per gene were converted to z-scores (mean-centred expression divided by standard deviation). Differential gene expression analysis was done using the linear models for microarray data (LIMMA [102]) package in R. In total, 17,403 protein coding genes were analysed. For each gene, mean expression level in the sensitive cell lines (*n* = 5) was compared to the mean expression level in resistant cell lines (*n* = 4). The lmFit function was used to fit robust linear models to the data and calculate mean expression. A moderated t-test was used to compare the expression between resistant and sensitive groups using the eBayes function. Resulting *p*-values were adjusted for multiple testing using false discovery rate (FDR). Significant differentially expressed genes were defined by FDR-adjusted *p*-value < = 0.05, and absolute log2 fold change >= 1.5. 

### 4.7. Mutational Signature Analysis

SNVs that pass confidence filtering and are present at MAF of 0.1% or less in gnomAD database, or confirmed somatic in COSMIC were considered somatic variants and selected for mutational signature analysis. Mutational signatures [103] were derived from the frequencies of all six types of single-base somatic substitutions of pyrimindine bases within a trinucleotide context (including the bases 5′ and 3′ of the mutated base). The R package deconstructSigs [104] was used to determine the contributions of known mutational signatures within individual cell lines using COSMIC SBS mutational signatures version 2 as reference.

## 5. Conclusions

These results implicate new genes as potential biomarkers of olaparib response from genomic data analyses of two independent groups of cancer cell lines. Using statistical methods we identify significant predictors of olaparib response based on gene expression, successfully validate some of these candidate genes by identifying genomic alterations (including sequence variants, copy number variants and differential expression) in olaparib-sensitive, -intermediate or -resistant HGSOC cell lines, and further highlight that promising validated genes have known relevant functions including interaction with PARP1 (*PUM3*, *EEF1A1*) or involvement in HR (*ELP4*). We present these results to encourage further experimental and clinical research into olaparib, and other PARPi, focusing on *PUM3*, *EEF1A1* and *ELP4* to investigate whether genomic and molecular alterations in these genes affect olaparib response in vitro, elucidate molecular mechanisms by which these genes contribute to olaparib response, and assess the potential utility of genomic alterations in these genes, in relation to *BRCA1/2* mutations and HR deficiency, for identifying patients most likely to benefit from olaparib treatment.

## Figures and Tables

**Table 1:** Candidate olaparib response genes derived from analyses of GDSC cell lines in known DNA repair and cell cycle pathways.

Gene	Ensembl ID	Analysis	Association
*APTX*	ENSG00000137074	Multivariate, univariate	sensitivity
*AURKB*	ENSG00000178999	univariate	sensitivity
*CCNA1*	ENSG00000133101	univariate	sensitivity
*CDC20*	ENSG00000117399	univariate	sensitivity
*CDKN2A*	ENSG00000147889	univariate	resistance
*CDKN2B*	ENSG00000147883	multivariate	resistance
*CDKN2C*	ENSG00000123080	univariate	resistance
*CKAP5*	ENSG00000175216	univariate	sensitivity
*E2F1*	ENSG00000101412	Multivariate, univariate	resistance
*EBP*	ENSG00000147155	univariate	resistance
*FANCE*	ENSG00000112039	Multivariate, univariate	sensitivity
*FXYD5*	ENSG00000089327	univariate	sensitivity
*GADD45G*	ENSG00000130222	univariate	resistance
*HMGA2*	ENSG00000149948	univariate	sensitivity
*IPO7*	ENSG00000205339	univariate	sensitivity
*KIF18A*	ENSG00000121621	univariate	sensitivity
*LLGL1*	ENSG00000131899	univariate	sensitivity
*MELK*	ENSG00000165304	univariate	sensitivity
*MNAT1*	ENSG00000020426	univariate	sensitivity
*ORC2*	ENSG00000115942	univariate	sensitivity
*PER1*	ENSG00000179094	multivariate	sensitivity
*PFN1*	ENSG00000108518	univariate	sensitivity
*PLK3*	ENSG00000173846	univariate	sensitivity
*PMS1*	ENSG00000064933	univariate	sensitivity
*PSMB6*	ENSG00000142507	univariate	sensitivity
*SLFN11*	ENSG00000172716	Multivariate, univariate	sensitivity
*STAG1*	ENSG00000118007	univariate	sensitivity
*TP53*	ENSG00000141510	univariate	sensitivity
*TUBA1C*	ENSG00000167553	univariate	sensitivity
*TUBA4A*	ENSG00000127824	univariate	resistance
*VAMP8*	ENSG00000118640	univariate	resistance
*XRCC5*	ENSG00000079246	univariate	sensitivity
*YWHAE*	ENSG00000108953	Multivariate, univariate	sensitivity

**Table 2:** Summary results from univariate analysis of GDSC cell lines for key candidate olaparib sensitivity genes.

Gene	Coefficient	95% Confidence Interval	FDR-Adjusted *p* Value
*PUM3*	−0.180	−0.247–−0.114	1.66 × 10^−4^
*ELP4*	−0.118	−0.191–−0.044	0.0311
*ELP5*	−0.131	−0.200–−0.063	0.00925
*EEF1A1*	−0.132	−0.210–−0.054	0.0211

**Table 3:** Frequency and types of GDSC cancer cell lines with mRNA gene expression data analysed in this study.

Cancer Type (TCGA Classification)	Abbreviation	Number of Cell Lines
Adrenocortical carcinoma	ACC	1
Acute lymphoblastic leukemia	ALL	22
Bladder Urothelial Carcinoma	BLCA	17
Breast invasive carcinoma	BRCA	45
Cervical squamous cell carcinoma and endocervical adenocarcinoma	CESC	13
Chronic Lymphocytic Leukemia	CLL	3
Colon adenocarcinoma and Rectum adenocarcinoma	COAD/READ	46
Lymphoid Neoplasm Diffuse Large B-cell Lymphoma	DLBC	30
Esophageal carcinoma	ESCA	32
Glioblastoma multiforme	GBM	34
Head and Neck squamous cell carcinoma	HNSC	39
Kidney renal clear cell carcinoma	KIRC	30
Acute Myeloid Leukemia	LAML	25
Chronic Myelogenous Leukemia	LCML	10
Brain Lower Grade Glioma	LGG	17
Liver hepatocellular carcinoma	LIHC	16
Lung adenocarcinoma	LUAD	57
Lung squamous cell carcinoma	LUSC	15
Medulloblastoma	MB	3
Mesothelioma	MESO	19
Multiple Myeloma	MM	16
Neuroblastoma	NB	25
Ovarian serous cystadenocarcinoma	OV	32
Pancreatic adenocarcinoma	PAAD	25
Prostate adenocarcinoma	PRAD	6
Small Cell Lung Cancer	SCLC	51
Skin Cutaneous Melanoma	SKCM	50
Stomach adenocarcinoma	STAD	20
Thyroid carcinoma	THCA	16
Uterine Corpus Endometrial Carcinoma	UCEC	9
Unknown	-	172

**Table 4:** Features of 18 HGSOC cell lines. The cell lines were derived from 12 HGSOC patients—14 from primary cases and 4 from recurrent (R) cases. All cases were of advanced stage (III-IV). Cell lines beginning with “TOV” are derived from tumor (*n* = 9) while those beginning with “OV” are derived from ascites (*n* = 9). “Pre-chemo” cell lines are derived from primary HGSOC cases that are completely naïve to any chemotherapy. “Post-chemo” cell lines are derived from primary HGSOC cases at the end of platinum-based chemotherapy, HGSOC cases at first recurrence [OV3133(R), TOV2295(R)], or HGSOC cases at second recurrence [OV866(2), OV1369(R2)].

Cell Line	Passage Number *	Response	Olaparib IC_50_ (µM) ± Standard Error of Mean (SEM)	Chemotherapy Status	Cellosaurus Number **
OV2295	P61	Sensitive	0.0003 ± 0.0004	Pre-chemo	CVCL_9T13
OV4453	P63		0.01 ± 0.0009	Pre-chemo	CVCL_9T20
TOV1946	P49		0.02 ± 0.007	Pre-chemo	CVCL_4062
TOV3041G	P52		0.02 ± 0.01	Post-chemo	CVCL_9T24
OV1946	P49		0.07 ± 0.05	Pre-chemo	CVCL_4375
TOV2978G	P67	Intermediate	0.45 ± 0.30	Pre-chemo	CVCL_9U73
TOV3133G	P65		0.58 ± 0.44	Pre-chemo	CVCL_4064
OV3133(R)	P71		0.75 ± 0.04	Post-chemo	CVCL_9T15
OV4485	P60		0.90 ± 0.58	Post-chemo	CVCL_9T21
TOV2295(R)	P57		1.52 ± 1.14	Post-chemo	CVCL_9T18
TOV3291G	P65		1.58 ± 0.23	Pre-chemo	CVCL_9T25
OV2295(R2)	P70		1.66 ± 0.99	Post-chemo	CVCL_9T14
TOV3133D	P66		2.00 ± 1.15	Post-chemo	CVCL_9T19
TOV2223G	P69		2.99 ± 1.20	Post-chemo	CVCL_4063
OV90	P63	Resistant	7.04 ± 2.33	Pre-chemo	CVCL_3768
OV866(2)	P108		8.11 ± 1.27	Post-chemo	CVCL_9T22
TOV1369	P65		9.02 ± 3.66	Pre-chemo	CVCL_9T17
OV1369(R2)	P66		21.71 ± 10.33	Post-chemo	CVCL_9T12

* Passage number at which each cell line was subjected to WES, SNP and gene expression arrays [17,29]. ** Expasy online knowledge resource on cell lines, web.expasy.org/cellosaurus (accessed on 29 January 2021).

## Data Availability

Data for GDSC cell lines are publicly available. In vitro olaparib response and mRNA gene expression data were downloaded from ftp://ftp.sanger.ac.uk/pub4/cancerrxgene/releases/release-7.0/v17.3_fitted_dose_response.xlsx (accessed on 8 July 2018) and https://www.synapse.org/#!Synapse:syn10463688/wiki/463140 (accessed on 7 June 2018) [73] respectively.

## Supplementary Materials

The following are available online at https://www.mdpi.com/2072-6694/13/6/1296/s1, Figure S1: Amplification of *CCNE1* locus in resistant OV866(2) (left) and intermediate TOV3291G (right) cell lines., Figure S2: Rare, potentially deleterious, homozygous variants in DNA repair and cell cycle genes for 18 HGSOC cell lines, Figure S3: Hierarchical clustering of cell lines by COSMIC single base substitution mutational signatures based on Euclidean distance and complete linkage, Figure S4: Distribution of in vitro olaparib response (IC_50_) across cell lines of multiple cancer types using TCGA classifications. Figure S5: Principal component analysis plot of GDSC cell lines based on gene expression. First two principal components (PC1 and PC2) are shown, Figure S6: Performance of elastic net multivariate linear regression model on prediction of IC_50_ in test data, Table S1: Candidate genes derived from multivariate analysis of GDSC pan-cancer cell lines, Table S2: Candidate genes derived from univariate analysis of GDSC pan-cancer cell lines, Table S3: Common candidate genes from multivariate and univariate analyses of GDSC pan-cancer cell lines, Table S4: List of DNA repair and cell cycle control genes investigated for genomic variations in HGSOC cell lines.
