# Supplementary material for: Candidate Markers of Olaparib Response from Genomic Data Analyses of Human Cancer Cell Lines
Source: Cancers (Basel). 2021 Mar 15;13(6):1296. doi: 10.3390/cancers13061296 (PMC7998846; doi:10.3390/cancers13061296)
Supplement: Supplementary file 1 [file cancers-13-01296-s001.zip › Supplementary.pdf]

Supplementary Information

Candidate Markers of Olaparib Response from Genomic Data Analyses of Human Cancer Cell Lines

Setor Amuzu, Euridice Carmona, Anne-Marie Mes-Masson, Celia M.T. Greenwood, Patricia N. Tonin, Ioannis Ragoussis

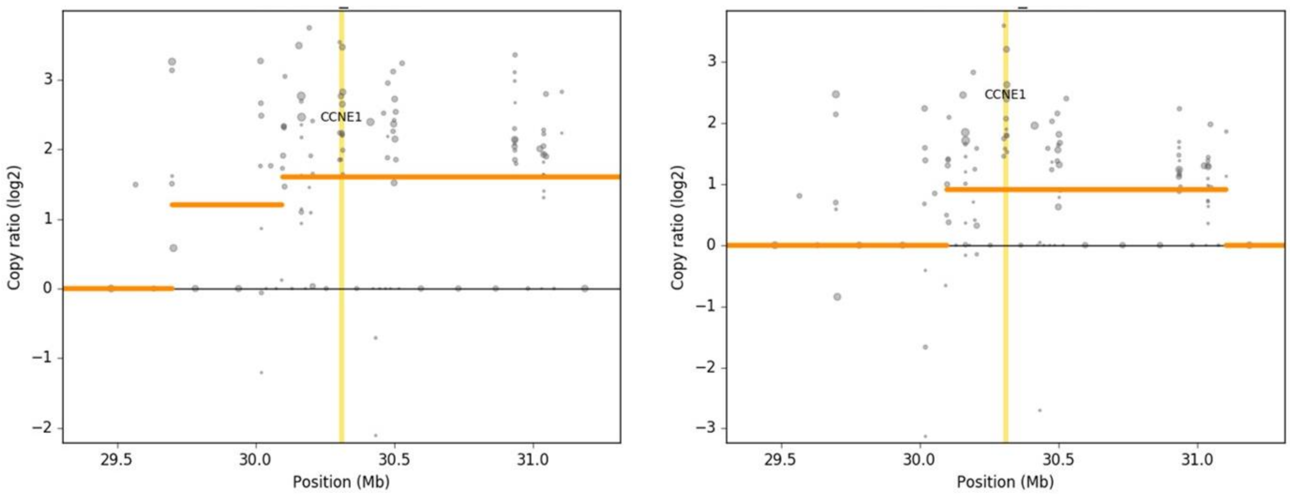

Figure S1. Amplification of *CCNE1* locus in resistant OV866(2) (left) and intermediate TOV3291G (right) cell lines.

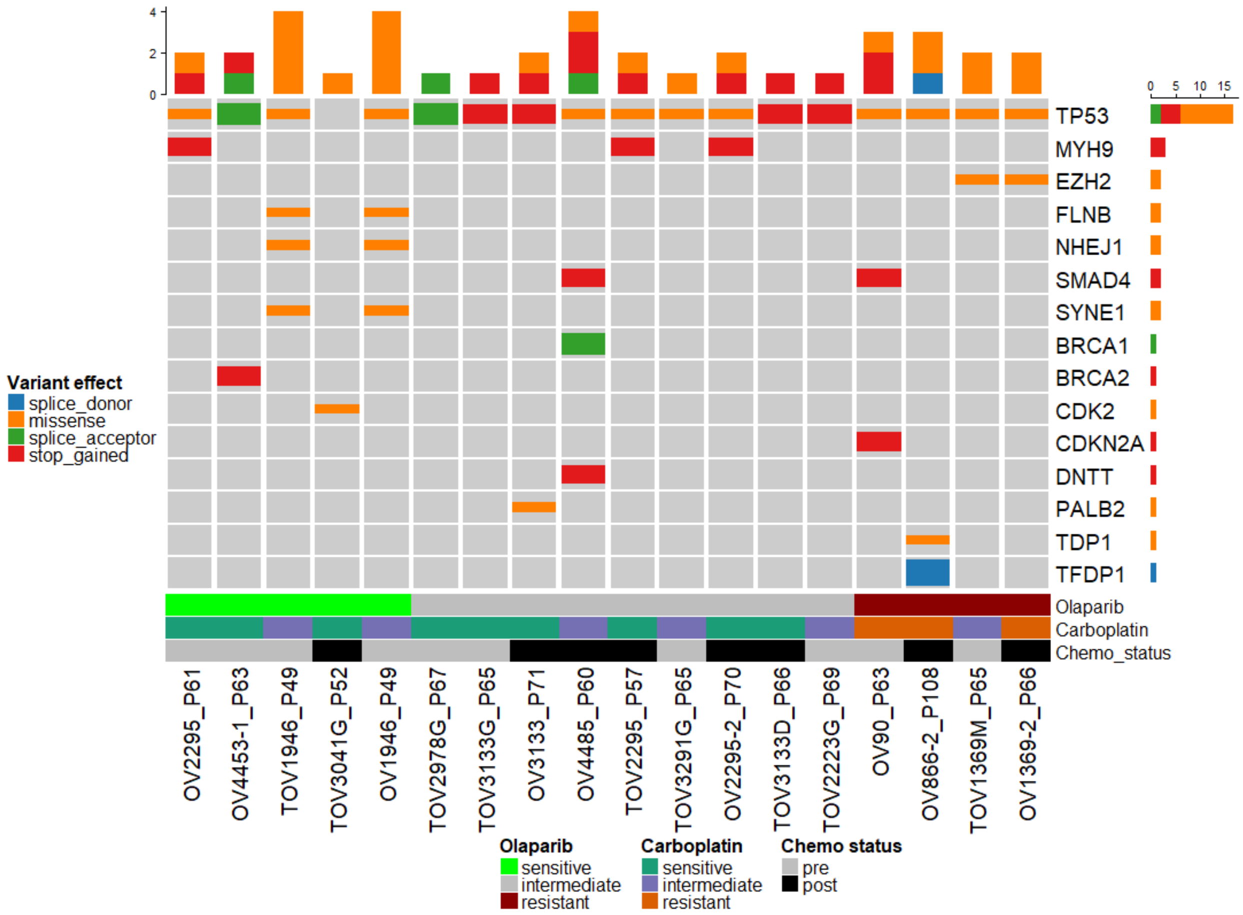

Figure S2. Rare, potentially deleterious, homozygous variants in DNA repair and cell cycle genes for 18 HGSOC cell lines. Cell lines arranged, left to right, in order of increasing resistance. OV2295 and OV1369(R2) are most sensitive and resistant cell lines respectively.

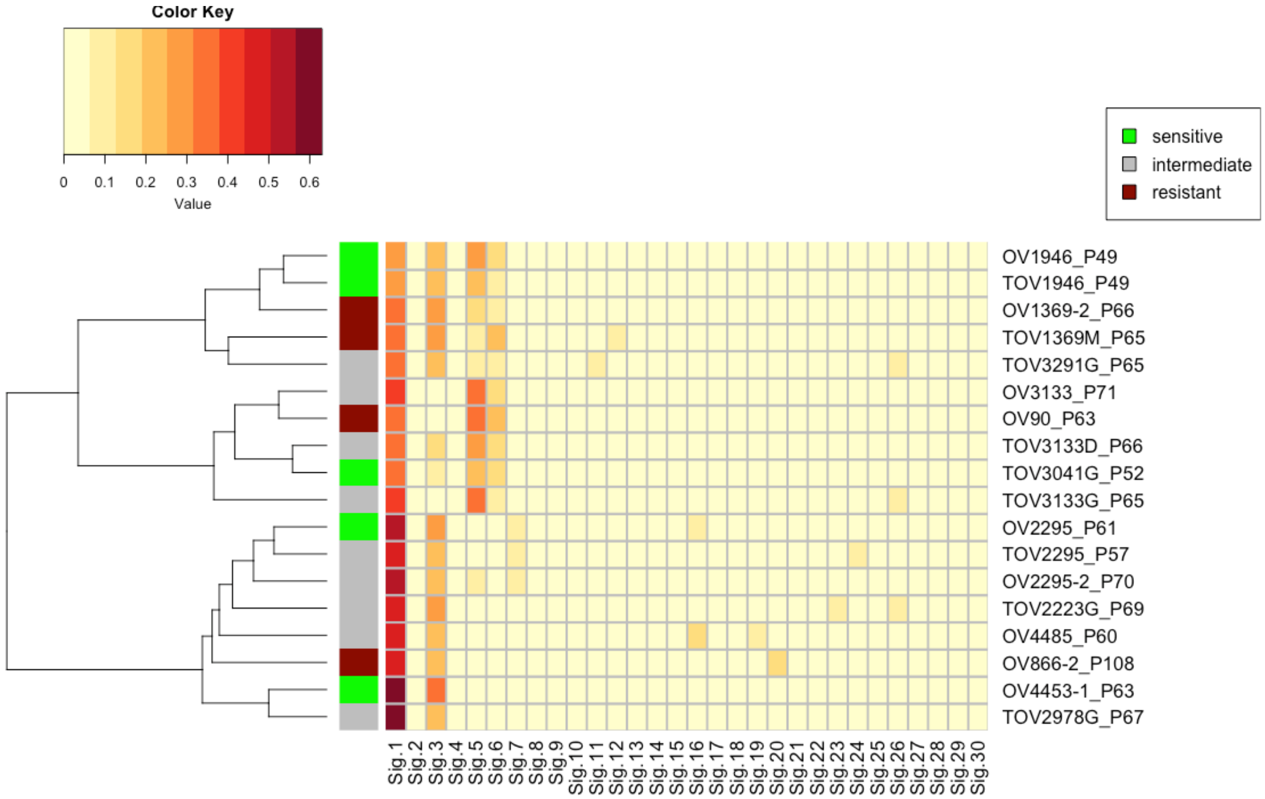

Figure S3. Hierarchical clustering of cell lines by COSMIC single base substitution mutational signatures based on Euclidean distance and complete linkage. Signature 1 is observed in all 18 cell lines while signature 3 is seen in 15 cell lines including *BRCA1*/2-mutated OV4485 and OV4453.

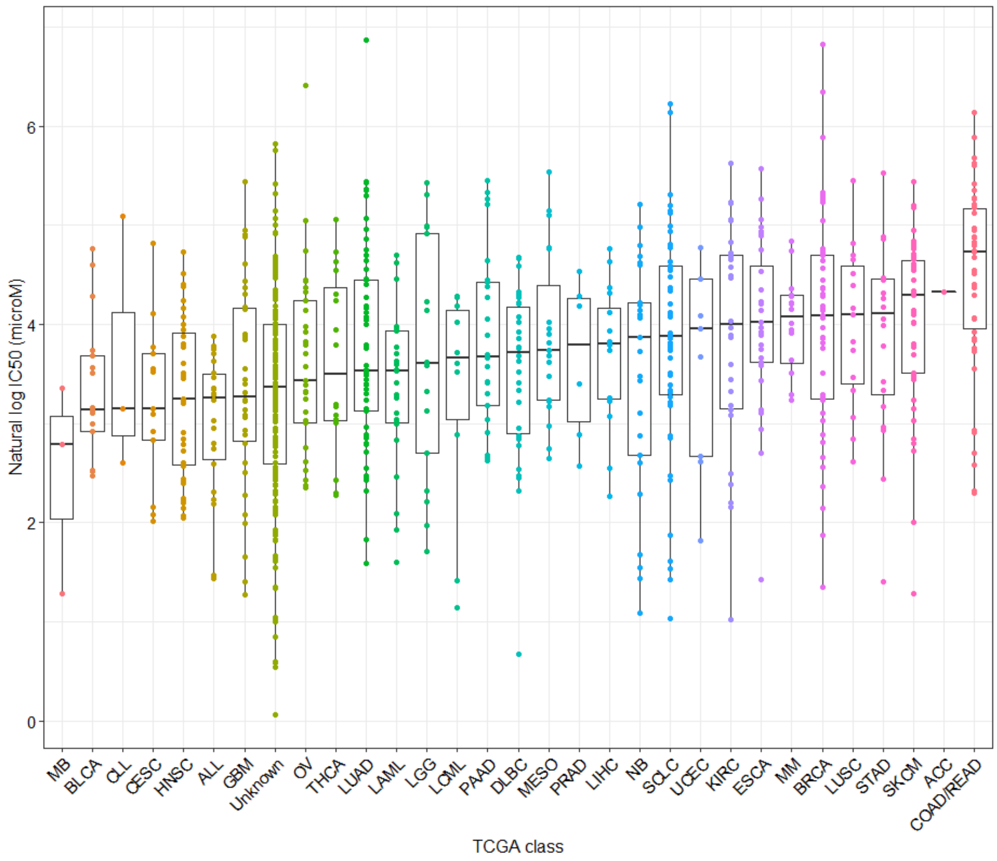

Figure S4. Distribution of *in vitro* olaparib response (IC_{50}) across cell lines of multiple cancer types using TCGA classifications. Dots represent individual cell lines. Boxplots represent cell lines in TCGA classes. Boxplots are ordered according to median IC_{50}.

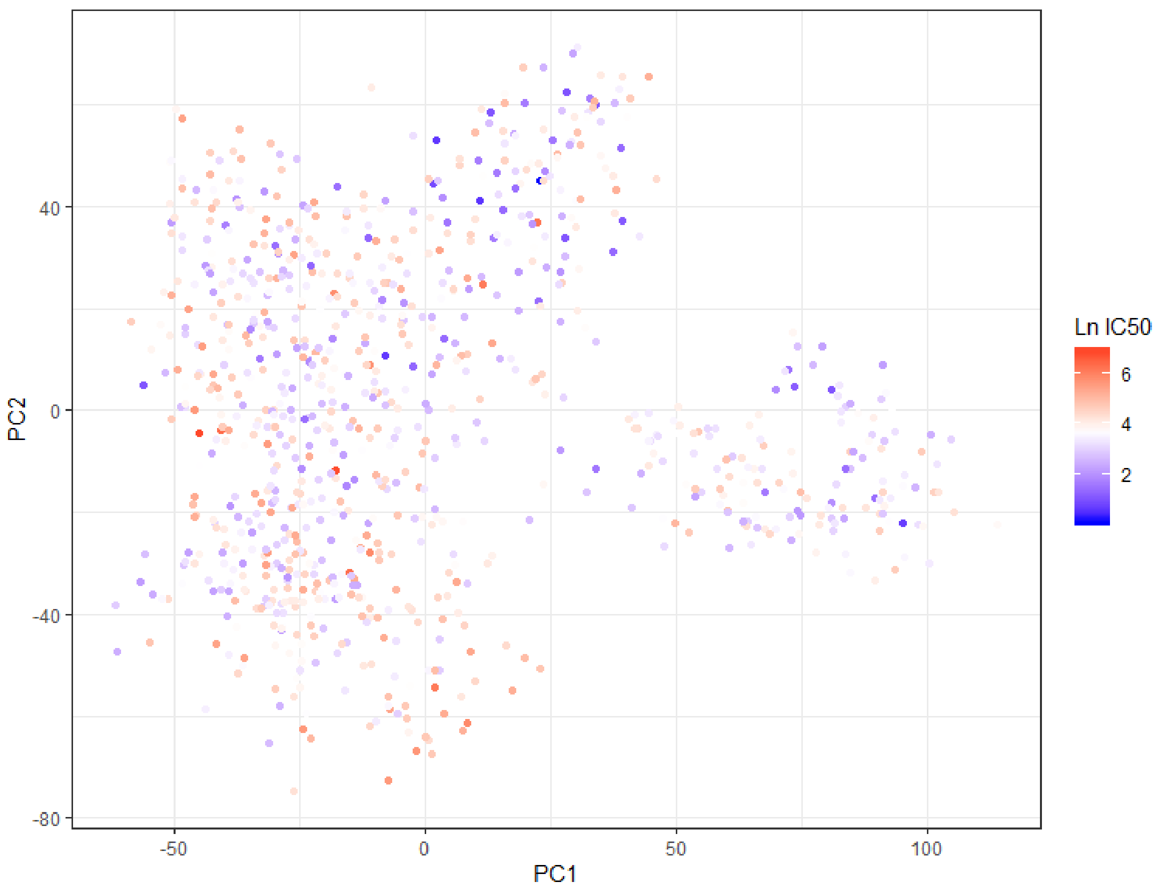

Figure S5. Principal component analysis plot of GDSC cell lines based on gene expression. First two principal components (PC1 and PC2) are shown. Each dot represents a cell line. PC1 and PC2 capture 16.7% of variation in cell lines. Dots are colored by natural log of olaparib IC_{50}.

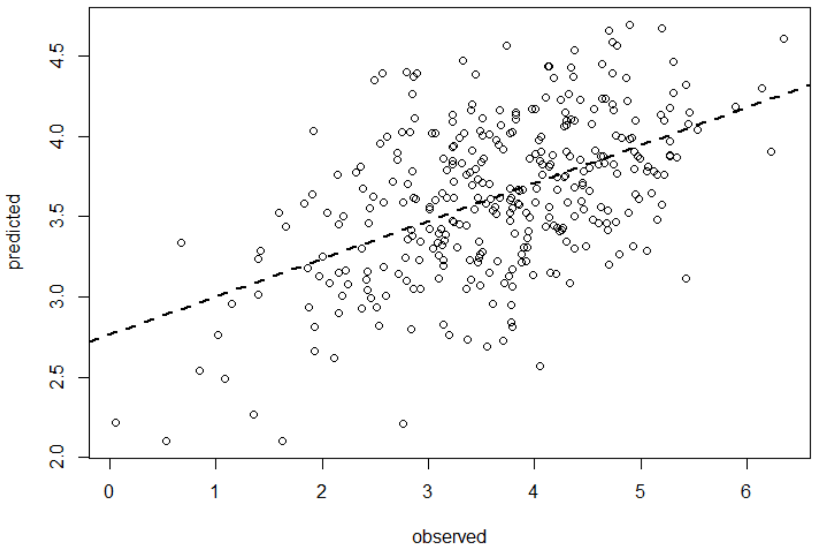

Figure S6. Performance of elastic net multivariate linear regression model on prediction of IC_{50} in test data. Observed IC_{50} values (horizontal axis) in test dataset plotted against predicted IC_{50} values (vertical axis) using elastic net model developed from training dataset. Points along the dashed line show agreement between observed and predicted values.

Tables S1–S3 in a separate file.

Table S4. List of DNA repair and cell cycle control genes investigated for genomic variations in HGSOC cell lines

		**Genes (*n* = 533)**		
ABL1	*CENPF*	*HES1*	*PAICS*	*SCARA3*
ACAT2	*CENPH*	*HEY1*	*PALB2*	*SETMAR*
AEN	*CENPK*	*HIST2H3C*	*PARG*	*SFN*
ALKBH1	*CENPM*	*HJURP*	*PARP1*	*SFRP1*
ANAPC1	*CENPN*	*HLTF*	*PARP2*	*SKP1*
ANAPC10	*CEP55*	*HMGA2*	*PARP3*	*SKP2*
ANAPC11	*CETN1*	*HMGB1*	*PARP4*	*SLC25A5*
ANAPC13	*CETN2*	*HMGB2*	*PARPBP*	*SLC38A2*
ANAPC2	*CETN3*	*HMGN2*	*PAXIP1*	*SLIRP*
ANAPC4	*CFL1*	*HMMR*	*PBK*	*SLX1A*
ANAPC5	*CFL2*	*HPRT1*	*PCNA*	*SLX1B*
ANAPC7	*CHAF1A*	*HSPD1*	*PER1*	*SMAD2*
ANLN	*CHAF1B*	*HUS1*	*PFN1*	*SMAD3*
ANP32E	*CHEK1*	*ID1*	*PFN2*	*SMAD4*
APC2	*CHEK2*	*IDH1*	*PKMYT1*	*SMARCA4*
APEX1	*CHFR*	*INCENP*	*PLK1*	*SMARCC1*
APEX2	*CIT*	*INF2*	*PLK3*	*SMC1A*

APTX	*CKAP2*	*IPO7*	*PMS1*	*SMC1B*
ASCC3	*CKAP5*	*ITGA6*	*PMS2*	*SMC2*
ASPM	*CKS1B*	*KAT5*	*PNKP*	*SMC3*
ATAD2	*CKS2*	*KIAA0101*	*POLA1*	*SMC5*
ATM	*CLK2*	*KIAA1524*	*POLB*	*SMC6*
ATR	*CREBBP*	*KIF11*	*POLD1*	*SMUG1*
ATRIP	*CSE1L*	*KIF15*	*POLD2*	*SNRPA1*
ATRX	*CTNNAL1*	*KIF18A*	*POLD3*	*SNRPD1*
AURKA	*CUL1*	*KIF20A*	*POLD4*	*SNRPD3*
AURKB	*CUL3*	*KIF22*	*POLE*	*SNRPG*
BABAM1	*CUL4A*	*KIF23*	*POLE2*	*SOX4*
BARD1	*CUL5*	*KIF3B*	*POLE3*	*SPAG5*
BCAS2	*DBF4*	*KIF4A*	*POLG*	*SPC24*
BCCIP	*DBI*	*KIFC1*	*POLH*	*SPC25*
BIRC5	*DCLRE1A*	*KNTC1*	*POLI*	*SPO11*
BLM	*DCLRE1B*	*KRT18*	*POLL*	*SSBP1*
BORA	*DCLRE1C*	*LIG1*	*POLM*	*STAG1*
BRCA1	*DCUN1D1*	*LIG3*	*POLQ*	*STAG2*
BRCA2	*DDB1*	*LIG4*	*PPP1CC*	*STK38L*
BRCC3	*DDB2*	*LIMK1*	*PPP1R12A*	*STK4*
BRIP1	*DERA*	*LLGL1*	*PPP2R1B*	*SYNE1*
BUB1	*DMC1*	*LLGL2*	*PPP2R2B*	*SYNE2*
BUB1B	*DNA2*	*MAD1L1*	*PPP2R5D*	*TCEA1*
BUB3	*DNTT*	*MAD2L1*	*PPP4C*	*TDG*
C17orf70	*DTL*	*MAD2L2*	*PPP4R1*	*TDP1*
C19orf40	*DTYMK*	*MAPK14*	*PPP4R4*	*TDP2*
C1orf86	*DUT*	*MAPRE1*	*PRDX4*	*TELO2*
CALM1	*E2F1*	*MAPRE2*	*PRDX6*	*TFDP1*
CCDC86	*E2F2*	*MASTL*	*PRIM1*	*TFDP2*
CCNA1	*E2F3*	*MBD4*	*PRKCA*	*TGFB1*
CCNA2	*E2F4*	*MCM2*	*PRKDC*	*TGFB2*
CCNB1	*E2F5*	*MCM3*	*PRPF19*	*TGFB3*
CCNB2	*E2F8*	*MCM4*	*PSMB3*	*TIPIN*
CCNB3	*EBP*	*MCM5*	*PSMB6*	*TK1*
CCNC	*ECT2*	*MCM6*	*PSMC3IP*	*TKT*
CCND1	*EEF1E1*	*MCM7*	*PTEN*	*TOP2A*
CCND2	*ENO1*	*MDC1*	*PTTG1*	*TOP3A*
CCND3	*EP300*	*MDM2*	*PTTG2*	*TOP3B*
CCNE1	*ERC1*	*MELK*	*RAB6A*	*TOPBP1*
CCNE2	*ERCC1*	*MGMT*	*RAB6C*	*TP53*
CCNF	*ERCC2*	*MKI67*	*RACGAP1*	*TP53BP1*
CCNG1	*ERCC3*	*MLH1*	*RAD1*	*TP73*
CCNG2	*ERCC4*	*MLH3*	*RAD17*	*TREX1*
CCNH	*ERCC5*	*MMS19*	*RAD21*	*TREX2*
CCNK	*ERCC6*	*MNAT1*	*RAD23A*	*TRIP13*
CCNL1	*ERCC8*	*MND1*	*RAD23B*	*TTK*
CCNL2	*ESPL1*	*MORF4L1*	*RAD50*	*TUBA1C*

CCNT1	*EXO1*	*MPG*	*RAD51*	*TUBA4A*
CCNT2	*EXO5*	*MRPL23*	*RAD51AP1*	*TUBG2*
CCT5	*EZH2*	*MRPL35*	*RAD51B*	*TUBGCP2*
CD320	*FAM64A*	*MRPL40*	*RAD51C*	*TUBGCP3*
CD9	*FAN1*	*MRPS17*	*RAD51D*	*TYMS*
CDC14A	*FANCA*	*MRPS28*	*RAD52*	*UBE2A*
CDC14B	*FANCC*	*MRTO4*	*RAD54B*	*UBE2B*
CDC16	*FANCE*	*MSH2*	*RAD54L*	*UBE2C*
CDC20	*FANCF*	*MSH3*	*RAD9A*	*UBE2N*
CDC20B	*FANCG*	*MSH6*	*RASSF1*	*UBE2S*
CDC23	*FANCI*	*MTHFD1*	*RB1*	*UBE2T*
CDC25A	*FANCL*	*MUS81*	*RBBP4*	*UBE2V2*
CDC25B	*FBL*	*MUTYH*	*RBBP8*	*UCK2*
CDC25C	*FBXO5*	*MYC*	*RBL1*	*UIMC1*
CDC26	*FDPS*	*MYH10*	*RBL2*	*UNG*
CDC27	*FEN1*	*MYH9*	*RBX1*	*UQCRH*
CDC45	*FLNA*	*MYL6*	*RECQL*	*USP1*
CDC5L	*FLNB*	*MYL7*	*RECQL4*	*VAMP8*
CDC6	*FMN1*	*MYLK*	*RECQL5*	*WDHD1*
CDC7	*FMN2*	*NABP2*	*REV1*	*WDR48*
CDCA5	*FOXN3*	*NBN*	*REV3L*	*WEE1*
CDCA8	*FXYD5*	*NCAPG*	*RFC1*	*WEE2*
CDH1	*FZR1*	*NDC80*	*RFC2*	*WRN*
CDK1	*GADD45A*	*NEIL1*	*RFC3*	*XAB2*
CDK10	*GADD45B*	*NEIL3*	*RFC4*	*XPA*
CDK2	*GADD45G*	*NEK2*	*RFC5*	*XPC*
CDK3	*GAPDH*	*NFATC2IP*	*RHOA*	*XRCC1*
CDK4	*GINS2*	*NHEJ1*	*RIF1*	*XRCC2*
CDK5	*GMNN*	*NSMCE4A*	*RIT1*	*XRCC3*
CDK6	*GSK3B*	*NTHL1*	*RMI1*	*XRCC4*
CDK7	*GSTO1*	*NUDT1*	*RNF4*	*XRCC5*
CDK8	*GTF2H1*	*NUDT15*	*RNF8*	*XRCC6*
CDK9	*GTF2H2*	*NUDT18*	*RNMT*	*YWHAB*
CDKL1	*GTF2H3*	*NUDT4*	*ROCK1*	*YWHAE*
CDKN1A	*GTF2H4*	*NUF2*	*ROCK2*	*YWHAG*
CDKN1B	*GTF2H5*	*OGG1*	*RPA1*	*YWHAH*
CDKN1C	*GTSE1*	*ORC1*	*RPA2*	*YWHAQ*
CDKN2A	*H2AFV*	*ORC2*	*RPA3*	*YWHAZ*
CDKN2B	*H2AFX*	*ORC3*	*RPA4*	*ZBTB17*
CDKN2C	*H2AFZ*	*ORC4*	*RRM1*	*ZRANB2*
CDKN2D	*HDAC1*	*ORC5*	*RRM2*	*ZW10*
CDKN3	*HDAC2*	*ORC6*	*RTEL1*	*ZWINT*
CENPE	*HERC2*	*PA2G4*		
